# High MHC-II expression in Epstein–Barr virus-associated gastric cancers suggests that tumor cells serve an important role in antigen presentation

**DOI:** 10.1038/s41598-020-71775-4

**Published:** 2020-09-08

**Authors:** Farhad Ghasemi, Tanner M. Tessier, Steven F. Gameiro, Allison H. Maciver, Matthew J. Cecchini, Joe S. Mymryk

**Affiliations:** 1 Department of Surgery, Western University, London, ON N6A 4V2 Canada; 2 Department of Microbiology and Immunology, Western University, London, ON N6A 3K7 Canada; 3 Department of Oncology, Western University, London, ON N6A 3K7 Canada; 4 Department of Pathology and Laboratory Medicine, Western University and London Health Sciences Centre, London, ON N6A 5C1 Canada; 5 Department of Otolaryngology, Head & Neck Surgery, Western University, London, ON N6A 5W9 Canada; 6 London Regional Cancer Program, Lawson Health Research Institute, London, ON N6C 2R5 Canada; 7 London Regional Cancer Program, Room A4-837, 790 Commissioners Rd. East, London, ON N6A 4L6 Canada

**Keywords:** Cancer, Gastrointestinal cancer, Tumour immunology, Tumour virus infections

## Abstract

EBV-associated gastric adenocarcinomas (EBVaGCs) often exhibit better clinical outcomes than EBV negative gastric cancers (GCs), which could be related to their consistent expression of foreign viral antigens. Antigen-presenting cells (APCs) present peptide antigens in the context of the class-II major histocompatibility complex (MHC-II). During inflammatory conditions, epithelial cells express MHC-II and function as accessory APCs. Utilizing RNA-seq data from nearly 400 GC patients, we determined the impact of EBV-status on expression of MHC-II components, genes involved in their regulation, and T-cell co-stimulation. Virtually all MHC-II genes were significantly upregulated in EBVaGCs compared to normal tissues, or other GC subtypes. Genes involved in antigen presentation were also significantly upregulated in EBVaGCs, as were the key MHC-II transcriptional regulators CIITA and RFX5. This was unexpected as the EBV encoded BZLF1 protein can repress CIITA transcription and is expressed in many EBVaGCs. Furthermore, MHC-II upregulation was strongly correlated with elevated intratumoral levels of interferon-gamma. In addition, expression of co-stimulatory molecules involved in T-cell activation and survival was also significantly increased in EBVaGCs. Thus, gastric adenocarcinoma cells may functionally contribute to the highly immunogenic tumor microenvironment observed in EBVaGCs via a previously unappreciated role in interferon-induced antigen presentation.

## Introduction

Epstein–Barr virus (EBV) was first identified from a case of Burkitt’s Lymphoma^1^. It is a gamma-herpesvirus that infects B-cells and mucosal epithelia, and induces cellular proliferation. EBV infections are highly prevalent and infection persists for the lifetime of the individual, likely because this large and complex virus possesses an extensive repertoire of immune evasion mechanisms^2^. In addition, EBV is responsible for multiple types of lymphomas, nasopharyngeal carcinomas, and EBV-associated gastric adenocarcinomas (EBVaGCs). Collectively, EBV infection is the causative agent for approximately 1.5% of all human cancers worldwide^1^.

The etiological role of EBV in gastric cancer (GC) was first identified in 1990^3^. It is currently estimated that EBV causes about 9% of GCs worldwide^4^. Importantly, EBVaGCs are a distinct molecular and clinical subtype of GC, exhibiting anatomical subsite preference in the stomach, male predominance, enhanced T-cell infiltration, widespread promoter hypermethylation, and improved prognosis as compared to other GC subtypes^4–8^.

A subset of viral genes are consistently expressed in EBV-associated cancers, including specific latency-associated factors, small virally encoded RNAs, and viral microRNAs (miRNAs)^1,9–11^. EBVaGCs express EBV nuclear antigen 1 (EBNA-1), and sensitive methods detect the mRNA expression of other latency-associated genes^9,10,12^. Importantly, mRNAs for a subset of EBV lytic genes, as well as many viral miRNAs are also frequently detected in EBVaGCs^10,12,13^. These transcripts are unlikely to represent contaminants from lytically infected B-cells infiltrating the tumor, as the lytic BZLF1 protein was detected immunohistochemically in EBVaGC cancer cells^14^ and the absolute score for immune cell infiltration was not significantly different between EBV-positive and EBV-negative GC samples^9^. EBV encoded miRNAs and proteins both functionally contribute to gastric carcinogenesis^15^. In addition to altering host cell growth, survival and signaling pathways, some viral genes are thought to help cancerous cells evade adaptive immunity and ensuing anti-tumor CTL responses^1,2,11,16^. As one example, EBV encoded miRNAs inhibit anti-viral CD4+ and CD8+ T-cell responses during primary infection of B-cells, which could similarly occur in EBV-associated cancers^17^.

To trigger an effective T-cell-specific anti-tumor response, a tumor-associated antigen must be presented in either the context of major histocompatibility complex class-I (MHC-I) or class-II (MHC-II)^18^. Surveilling antigen-presenting cells (APCs) initially acquire specific antigenic peptides. These exogenous peptides are subsequently presented in the context of MHC-II on the APC cell surface to activate antigen-specific CD4+ helper T-cells^19^. In addition to crosslinking of the antigen-MHC-II complex with its cognate T-cell receptor (TCR), T-cell activation requires the ligation of co-stimulatory molecules between the APC and T-cell. This two step process provides the necessary signals to trigger proliferation and survival of antigen specific T-cells^20^. Activated CD4+ T-cells subsequently stimulate CD8+ cytotoxic T-cells (CTLs) that similarly recognize the same peptide antigen. Activated CTLs can then target and lyse tumor cells displaying that specific, endogenously derived antigenic peptide in the context of cell surface MHC-I^21,22^.

We and others have previously reported that EBVaGCs express higher levels of MHC-I than other GC subtypes^23,24^. Thus, EBVaGCs may more effectively display endogenously derived viral or neo-antigenic peptides, enhancing their lysis by CTLs. As mentioned above, presentation of viral antigens to activate CD4+ helper T-cells occurs in the context of MHC-II molecules, which are primarily expressed by professional APCs, such as dendritic cells (DCs), macrophages, and B-cells^25^. However, exposure of epithelial cells to pro-inflammatory cytokines like interferon-gamma (IFNγ) induces expression of MHC-II. These epithelial cells can subsequently function as accessory APCs to present antigens and stimulate an effective CTL response^26^. Increased levels of MHC-II proteins on epithelial cells should enhance the presentation of exogenously derived viral and tumor specific peptide antigens to generate enhanced CTL responses. Indeed, the underappreciated role for tumor cell derived MHC-II in anti-tumor immunity is becoming apparent, with numerous reports suggesting that tumor-specific MHC-II expression is correlated with favorable outcomes in many cancer types, including GCs^27,28^. Interestingly, the product of the EBV lytic gene BZLF1, which is expressed in many EBVaGCs^10,12–14,29–31^, is known to interfere with MHC-II gene expression and function in other contexts, and could potentially contribute to immune evasion in EBVaGCs^32–34^.

In this study, we used RNA-sequencing data from nearly 400 human GCs to comprehensively assess if EBV presence altered expression of genes involved in the MHC-II pathway and associated epithelial APC function. EBVaGC tumors exhibited significantly upregulated expression of virtually all MHC-II genes compared to other GC subtypes or normal control tissue. Similarly, EBVaGC tumors exhibited significantly increased expression of genes encoding necessary antigen loading and presentation components. Importantly, these inducible MHC-II genes were expressed at levels that were orders of magnitude higher than genes specifically associated with professional APCs, making it unlikely that these increases were related to inordinate infiltration by those types of APCs. In addition, EBVaGC samples exhibited significant upregulation of master regulators of the MHC-II transcriptional control system, including class-II major histocompatibility complex transactivator (CIITA) and regulatory factor X5 (RFX5)^26^. The higher intratumoral levels of IFNγ observed in EBVaGC tumors was highly correlated with coordinated increases in the mRNA levels of MHC-II antigen presentation pathway genes. In addition, EBVaGCs exhibited significantly upregulated levels of T-cell co-stimulatory genes encoding factors involved in T-cell activation and survival compared to other GC subtypes and normal control tissue. In combination, these results indicate that gastric adenocarcinoma cells likely contribute to the highly immunogenic tumor microenvironment observed in EBVaGCs by playing a previously unappreciated role in interferon-induced MHC-II dependent antigen presentation. Importantly, these results identify profound differences in the immune landscape between the tumor microenvironments of EBVaGCs and other GC subtypes, which may contribute to the improved survival associated with EBVaGCs^8^ and their dramatic responsiveness to immune checkpoint inhibitors such as pembrolizumab^35^.

## Results

### EBVaGCs express higher levels of MHC class II α- and β-chain genes

**Figure 1:**
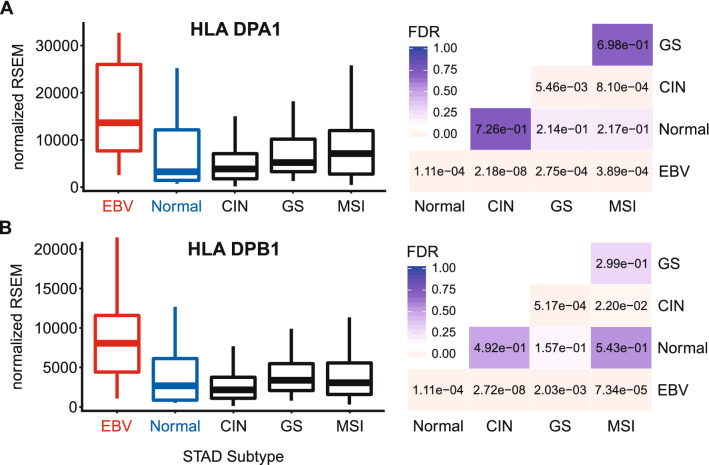
Expression of the DP MHC-II α- and β-chain genes in GC subtypes and normal gastric tissue. False discovery rate (FDR) adjusted p-values for each statistical comparison are shown on the right. Figure was generated using the ggplot2 package in RStudio (version 1.2.1335; https://rstudio.com) and final figure layouts were adjusted with CorelDRAW (version X7).

**Figure 2:**
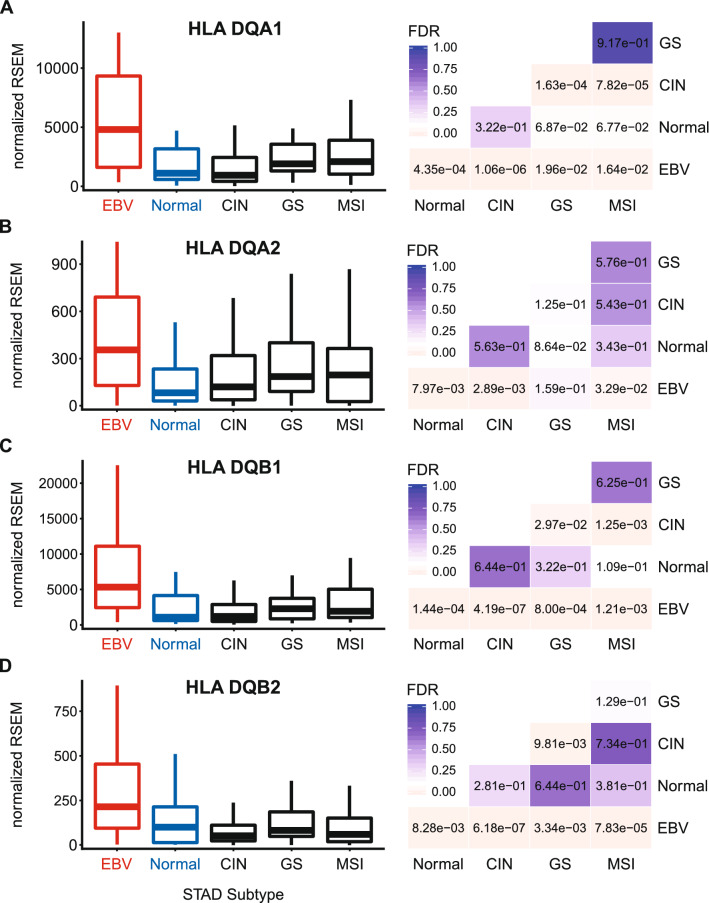
Expression of the DQ MHC-II α- and β-chain genes in GC subtypes and normal gastric tissue. FDR adjusted p-values for each statistical comparison are shown on the right. Figure was generated using the ggplot2 package in RStudio (version 1.2.1335; https://rstudio.com) and final figure layouts were adjusted with CorelDRAW (version X7).

**Figure 3:**
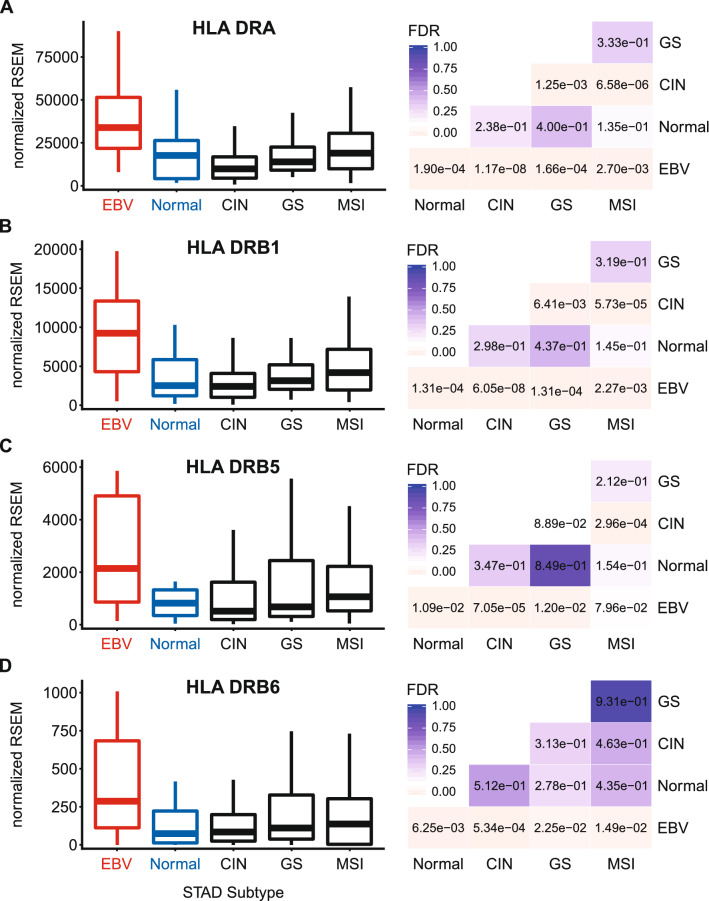
Expression of the DR MHC-II α- and β-chain genes in GC subtypes and normal gastric tissue. FDR adjusted p-values for each statistical comparison are shown on the right. Figure was generated using the ggplot2 package in RStudio (version 1.2.1335; https://rstudio.com) and final figure layouts were adjusted with CorelDRAW (version X7).

**Figure 4:**
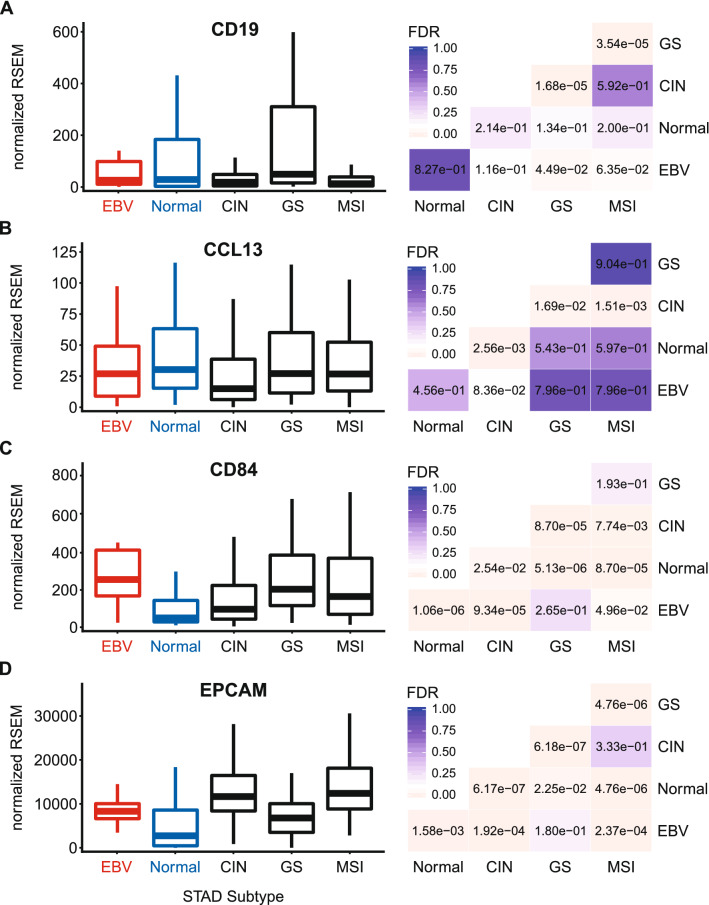
Comparison of expression of APC and epithelia markers in GC subtypes and normal gastric tissue. Relative expression levels of genes associated with APCs (**A**–**C**) or epithelial cells (**D**). FDR adjusted p-values for each statistical comparison are shown on the right. Figure was generated using the ggplot2 package in RStudio (version 1.2.1335; https://rstudio.com) and final figure layouts were adjusted with CorelDRAW (version X7).

Constitutive MHC-II expression is primarily restricted to professional APCs—DCs, B-cells, and macrophages^25^. However, exposure to pro-inflammatory cytokines can induce MHC-II molecule expression in non-immune cells, such as those of the gastric epithelia^36–40^. The three polymorphic MHC-II molecules HLA-DP, HLA-DQ, and HLA-DR exist as heterodimers comprised of α- and β-chains^19^. All three of these MHC-II protein complexes have been detected in GC tumor cells by immunohistochemical analyses^40^, and their expression is correlated with improved prognosis. Several immunohistochemical studies have similarly shown significant expression of HLA-DR specifically in EBVaGCs and concluded that EBV-positive tumor cells are more likely to express HLA-DR than their EBV-negative counterparts^41,42^. Using mRNA expression data from the Cancer Genome Atlas (TCGA) GC cohort, we initially assessed the impact of EBV status on expression of the HLA-DPA1, -DPB1, -DQA1, -DQA2, -DQB1, -DQB2, -DRA, -DRB1, -DRB5, and -DRB6 genes. These encode the α- and β-chains for all three isotypes (Figs. 1, 2, 3). With the exception of HLA-DQA2 in the GS subtype, all EBVaGC patient samples expressed significantly higher levels of mRNA for all 10 MHC-II genes analyzed compared to most other GC subtypes or normal control tissues. This is in good agreement with previous results reported for HLA-DR by immunohistochemistry^41,42^. Thus, EBVaGCs express significantly higher levels of MHC-II mRNA versus other GC subtypes or normal control tissues. It is noteworthy that based on the normalized read levels, all of these genes are expressed at levels 10 to 100 times higher than markers of professional APCs, such as CD19 (B-cells), CCL13 (macrophages), and CD84 (DCs)^43–45^ (Fig. 4A–C). However, these normalized read levels are comparable to that of an established GC epithelial marker, epithelial cell adhesion molecule (EPCAM)^46^ (Fig. 4D). Thus, based on the magnitude of expression of the MHC-II α- and β-chains, it is likely that they are being expressed by GC cells, rather than infiltrating professional APCs. This is supported by immunohistochemical analyses reporting expression of MHC-II molecules, particularly HLA-DR, by most GC epithelial cells^40^, including EBVaGCs^41,42^. A very recent single cell RNA sequencing study reported that malignant epithelial cells from an EBVaGC expressed higher levels of both HLA-DPA1 and HLA-DPB1 compared to the other GCs studied, definitively showing that these mRNAs originate from carcinoma cells, rather than professional APCs within the tumor^47^.

### EBVaGCs express higher levels of genes encoding key components of the MHC-II antigen presentation pathway

**Figure 5:**
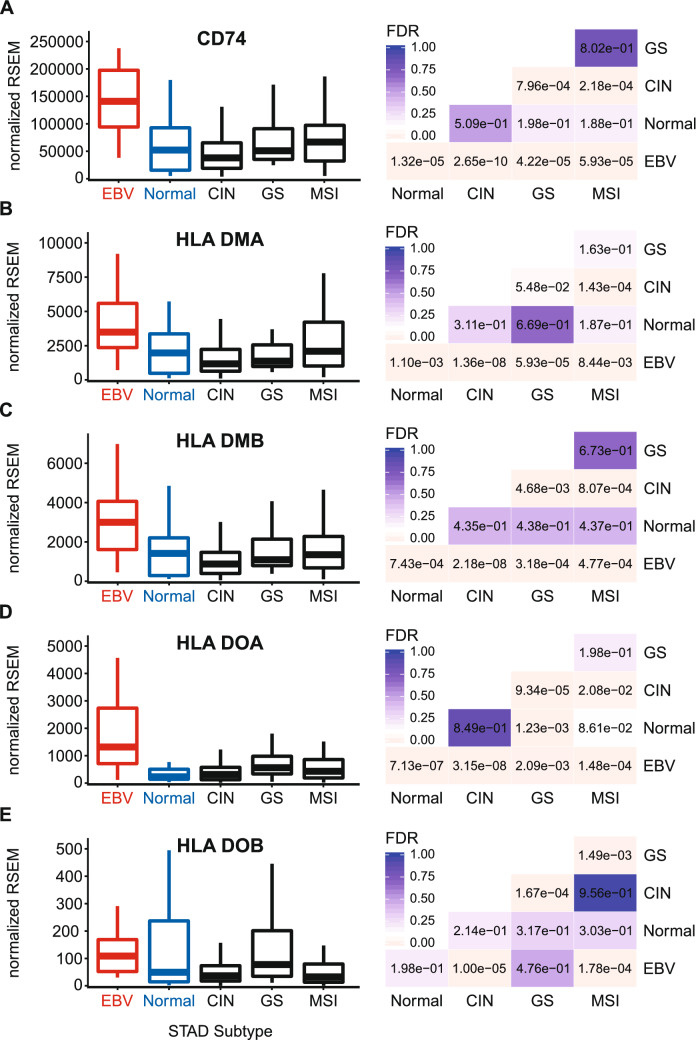
Expression of the invariant chain and MHC class II-like genes in GC subtypes and normal gastric tissue. FDR adjusted p-values for each statistical comparison are shown on the right. Figure was generated using the ggplot2 package in RStudio (version 1.2.1335; https://rstudio.com) and final figure layouts were adjusted with CorelDRAW (version X7).

Newly synthesized MHC-II α- and β-chains form a trimeric complex in the endoplasmic reticulum with a non-polymorphic protein called the invariant chain (Ii). This is encoded by the Cluster of Differentiation 74 (CD74) or HLA-DR antigen-associated invariant chain gene^48^. Interaction with the Ii chain blocks loading with endogenously derived peptides and directs the Ii-MHC-II complex to the endosomal-lysosomal antigen-processing compartments, which contain exogenously derived antigenic peptides^19^. Proteolytic cleavage of Ii generates the class II-associated invariant chain peptide (CLIP) which remains in the peptide-binding groove. Like the MHC-II α- and β-chain genes, EBVaGCs exhibited significantly upregulated CD74 mRNA expression compared to other GC subtypes or normal control tissues (Fig. 5A).

CLIP is removed by the MHC class II-like heterodimer, HLA-DM, which allows loading with lysosomally generated antigenic peptides^49^. Antigenic peptide binding is further regulated by HLA-DO, another MHC class II-like heterodimer, which influences the activity of HLA-DM^50^. These dimeric class II-like molecules are encoded by the HLA-DMA, HLA-DMB, HLA-DOA, and HLA-DOB genes. Expression of all four of these genes are upregulated in EBVaGCs compared to other GC subtypes or normal control tissues (Fig. 5B–E). This global upregulation of genes encoding the MHC-II invariant chains and class-II like genes in EBVaGCs suggests that all necessary components of the MHC-II antigen presentation pathway are expressed in EBVaGCs at significantly higher levels than observed in other GC subtypes or normal control tissues. Furthermore, the very high numerical level of normalized expression of all these genes, except for HLA-DOB, are likely indicative of expression by the actual adenocarcinoma cells within the tumor.

### EBVaGCs express higher levels of transcriptional regulators of MHC-II gene expression

**Figure 6:**
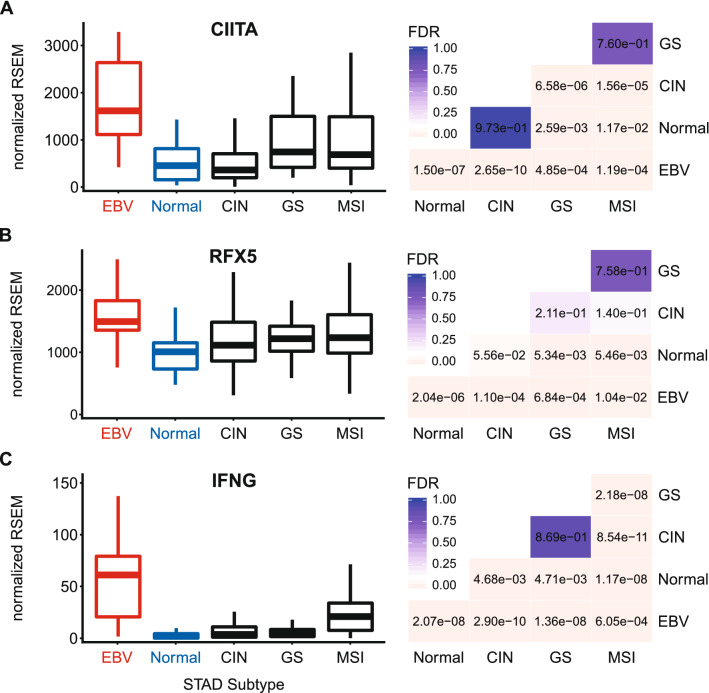
Expression of CIITA, RFX5, and IFNG mRNA in GC subtypes and normal gastric tissue. FDR adjusted p-values for each statistical comparison are shown on the right. Figure was generated using the ggplot2 package in RStudio (version 1.2.1335; https://rstudio.com) and final figure layouts were adjusted with CorelDRAW (version X7).

Transcriptional control of the MHC-II antigen presentation pathway is completely dependent on the master transcriptional regulator CIITA^26,51^. Consistent with the high levels of MHC-II genes and related genes, significantly higher levels of CIITA were present in EBVaGC samples compared to other GC subtypes or normal control tissues (Fig. 6A). In addition, higher levels of RFX5—another critical transcriptional regulator of MHC-II genes^26^—were also detected in EBVaGC samples compared to other GC subtypes or normal control tissues (Fig. 6B).

As mentioned above, many non-hematopoietic cells, including gastric epithelial cells, can be stimulated by IFNγ to express MHC-II dependent antigen presentation pathway components^26,36–40^. Analysis of the IFNγ gene (IFNG) mRNA levels revealed that it was expressed at significantly higher levels in EBVaGCs compared to other GC subtypes or normal control tissues (Fig. 6C). Although the relative levels of IFNG mRNA are low, their normalized numerical values were of similar magnitude to other leukocyte specific genes (compare Figs. 4A–C and 6C).

### Expression of most genes in the MHC-II pathway is coordinately upregulated in EBVaGCs

**Figure 7:**
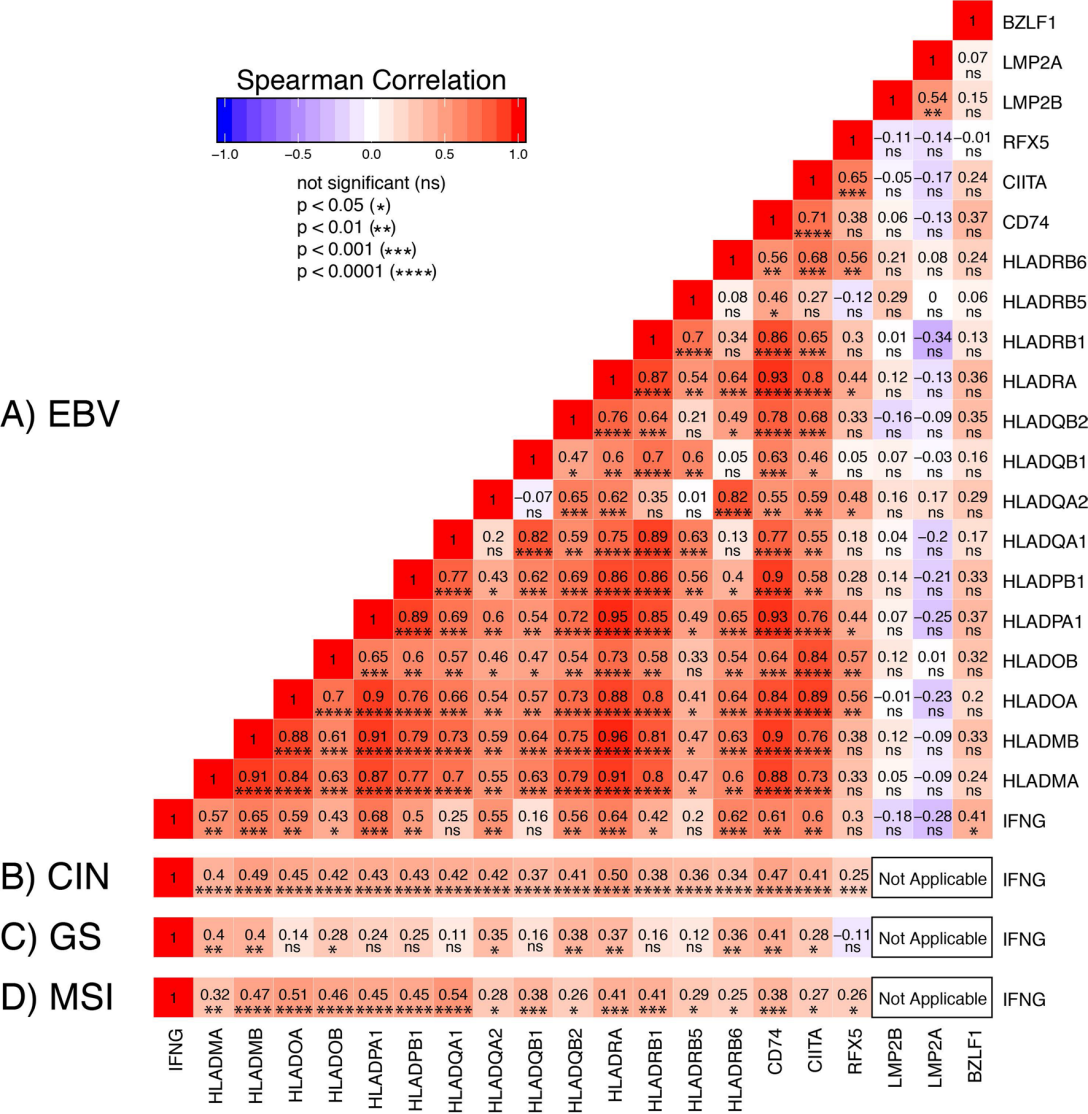
Correlation matrix of genes involved in the MHC-II antigen presentation pathway in GC subtypes. Heatmap of Spearman correlation analysis of mRNA expression of the indicated MHC-II pathway genes in EBVaGC (**A**). Comparisons with EBV genes reported to antagonize interferon-γ response are also shown. Spearman correlations between mRNA levels for IFNG and MHC-II pathway genes are also shown for the CIN (**B**), GS (**C**), and MSI (**D**) subtypes. Numbers in boxes indicate Spearman’s rank correlation coefficient of analyzed gene pairs and p-values. Figure was generated using the ggplot2 package in RStudio (version 1.2.1335; https://rstudio.com) and final figure layouts were adjusted with Adobe Illustrator (version CS6).

To further investigate the relationship between IFNγ expression and upregulation of MHC-II pathway genes, we generated a correlation matrix for the EBVaGC samples (Fig. 7A). In each patient sample, the expression levels of nearly all MHC-II antigen presentation-specific genes was statistically correlated in a pairwise fashion. In particular, the expression of all but 4 of these genes were highly and significantly correlated with IFNG levels (Fig. 7A). Thus, exposure to inflammatory cytokines like IFNγ is likely responsible for the upregulated expression of CIITA, RFX5, and subsequent expression of all the MHC-II genes and related genes required for antigen loading and presentation observed in EBVaGCs. In addition, the correlation matrix clearly demonstrates the simultaneous coordination of the MHC-II transcriptional control system dictated by CIITA, the IFNγ inducible master regulator^26,51^. Similarly, mRNA levels of all MHC-II pathway genes were correlated with IFNG in the CIN and MSI GC subtypes (Fig. 7B,D). Interestingly, fewer correlations were present in the GS subtype, which could reflect the low levels of IFNG mRNA in those samples (Fig. 6C).

### Immune infiltration is correlated with higher levels of most MHC-II pathway components in EBVaGCs

Multiple immunohistochemistry based studies have concluded that higher expression of HLA-DR protein by carcinoma tissue in GCs^28^, including EBVaGCs specifically^41,42^, is directly correlated with lymphocytic infiltration. High-quality diagnostic histologic slides were available for 24 of the EBVaGCs from the TCGA cohort (see Supplementary Figure S1 online). Based on these slides, the fraction of inflammatory cells from each sample was estimated using QuPath^52^ (see Supplementary Table S1 online) and this was correlated with expression of each MHC-II pathway component (Supplementary Table S2 online). Despite the small sample size, clear correlations were observed between immune infiltration and the expression of the majority of MHC-II pathway genes. Although above the FDR cutoff of ≤ 0.1, many of the other MHC-II gene comparisons approached significance. These results support the conclusions by others based on immunohistochemical analysis of HLA-DR protein levels^41,42^, extend them to include many additional MHC-II pathway genes, and provide further support for a key role of lymphocyte derived pro-inflammatory cytokines like IFNγ in upregulation of MHC-II expression in EBVaGCs.

### EBV encoded antagonists of the IFNγ response pathway do not inhibit MHC-II upregulation in EBVaGCs

EBV encodes multiple antagonists of the IFNγ response pathway, including latent membrane protein 2A and 2B (LMP-2A and LMP-2B, respectively), and BZLF1^53,54^. The genes encoding each of these viral proteins are expressed at variable levels in EBVaGCs^10,12–14,29–31^. To determine if the expression of these viral genes were inversely correlated with the IFNγ response, we expanded our pairwise comparison to include LMP2A, LMP2B, and BZLF1. We did not find a significant inverse correlation between these genes and any of the MHC-II pathway genes (Fig. 7A). Thus, despite the relatively high levels of expression of these EBV encoded transcripts in many EBVaGCs^10^, they do not appear to be effective antagonists of IFNγ-dependent transcriptional activation of MHC-II pathway genes in these human tumors.

### EBVaGCs express higher levels of T-cell co-stimulatory molecules

**Figure 8:**
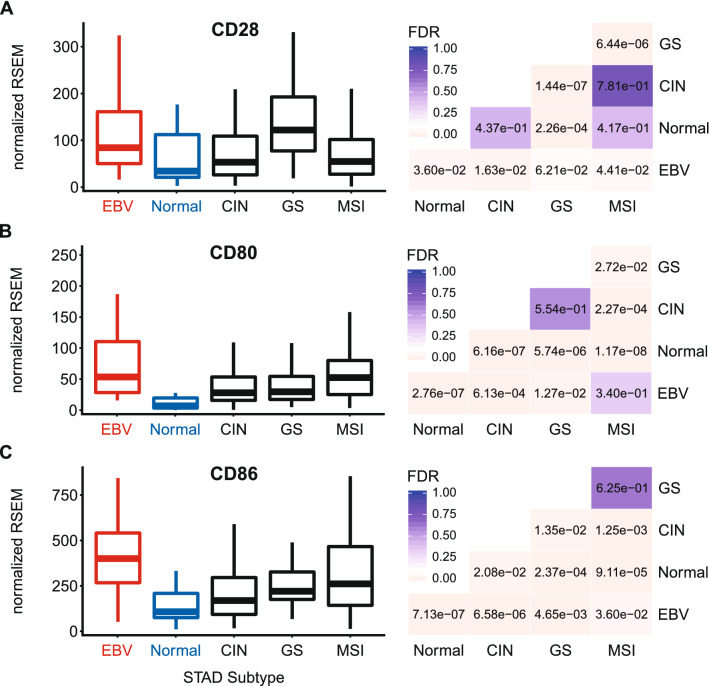
Expression of genes encoding the T-cell co-stimulatory molecules CD28, CD80, and CD86 in GC subtypes and normal gastric tissue. FDR adjusted p-values for each statistical comparison are shown on the right. Figure was generated using the ggplot2 package in RStudio (version 1.2.1335; https://rstudio.com) and final figure layouts were adjusted with CorelDRAW (version X7).

T-cell activation requires co-stimulation via ligation of the constitutively expressed CD28 receptor with either CD80 or CD86 expressed on APCs^20^. Utilizing the mRNA expression data, EBVaGCs expressed higher levels of CD28 compared to other GCs or normal control tissues, suggesting T-cell infiltration (Fig. 8A). Similarly, EBVaGCs expressed both CD80 and CD86 at significantly increased levels compared to other GCs and normal control tissues, with the exception of CD80 in the MSI subtype (Fig. 8B,C). Thus, like the MHC-II pathway genes, co-stimulatory molecules necessary for APC-dependent activation of T-cells are similarly present at higher levels in EBVaGCs.

### EBVaGCs express higher levels of inducible T-cell survival signal molecules

**Figure 9:**
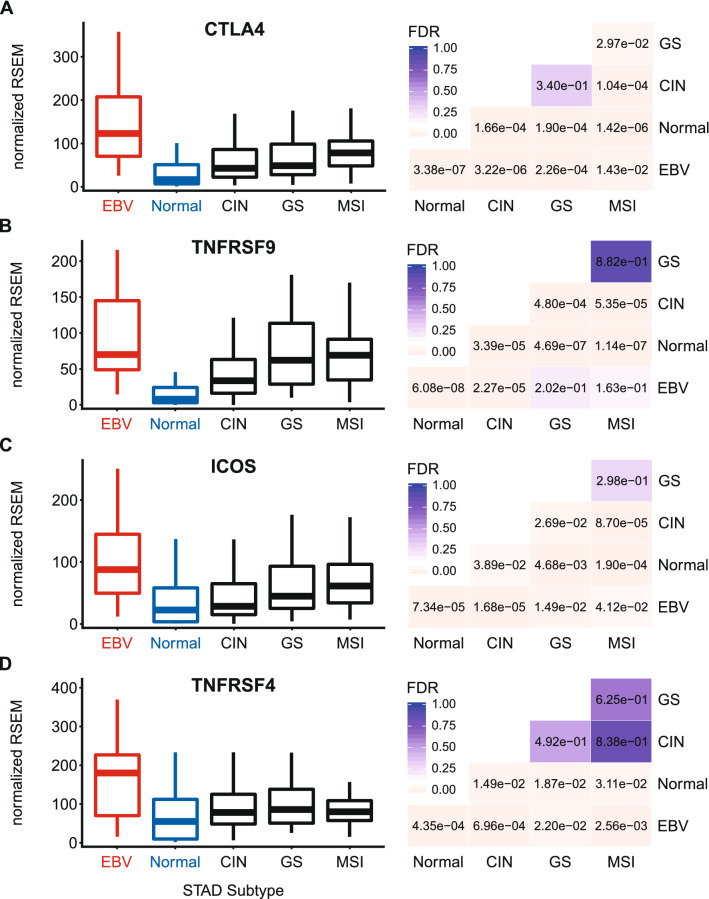
Expression of inducible T-cell survival signal molecules CTLA4, TNFRSF9, ICOS, and TNFRSF4 in GC subtypes and normal gastric tissue. FDR adjusted p-values for each statistical comparison are shown on the right. Figure was generated using the ggplot2 package in RStudio (version 1.2.1335; https://rstudio.com) and final figure layouts were adjusted with CorelDRAW (version X7).

The presence of enhanced MHC-II dependent antigen presentation in EBVaGCs would predict increased T-cell activation. Utilizing the RNA-seq GC dataset, we found that the mRNA levels of CD152, which encodes CTLA-4, a marker of T-cell activation^55^, was significantly upregulated in EBVaGCs compared to other GC subtypes and normal control tissues (Fig. 9A). We also looked at genes encoding inducible, T-cell activation-dependent, survival signal molecules and their respective ligands^20,56^. EBVaGCs expressed higher levels of CD137 (4-1BB, TNFRSF9) compared to normal control tissues, but not compared to several of the other GC subtypes (Fig. 9B). Next, we looked at the genes that encode for the inducible T-cell co-stimulator (ICOS), which was also significantly upregulated in EBVaGCs compared to other GC subtypes and normal control samples (Fig. 9C). Similarly, EBVaGCs expressed higher levels of OX40 (TNFRSF4, CD134) compared to other GC subtypes and normal control samples (Fig. 9D). The increased expression of these indices of T-cell activation, which are only induced following TCR-mediated antigen-specific T-cell activation and/or CD28 co-stimulation^20,56^, indicates that T-cells are generally activated and proliferating within the EBVaGC tumor microenvironment at higher levels compared to other GC subtypes and normal control tissues. This is fully consistent with increased MHC-II dependent presentation of tumor antigens.

## Discussion

EBV-associated cancers express viral latency genes, but also frequently express additional viral genes involved in the lytic phase of infection^1^. Many of these viral effectors contribute to immune evasion during infection by blocking antigenic peptide presentation, and could similarly enhance evasion of anti-tumor responses if expressed in cancers. In contrast, detection of foreign antigenic peptides derived from viral proteins could lead to a more effective anti-cancer immune response and improved patient survival.

The MHC-II apparatus represents a key component of adaptive anti-tumor immunity. MHC-II is constitutively expressed on the surface of professional APCs. By sampling their local environment via phagocytosis, APCs acquire antigens and process them for presentation to CD4 + T-cells in the context of MHC-II^19^. Ligation of the antigen-MHC-II complex with the CD4 + TCR initiates T-cell activation that, in the presence of co-stimulatory signals, ideally leads to an effective adaptive immune response against external or internal threats, including malignant cells and infectious agents^20^.

In epithelial cells, the pro-inflammatory cytokine IFNγ can induce MHC-II expression^26,36–39^. This coordinated induction of the MHC-II pathway apparatus in epithelial cells allows them to function as accessory APCs, accentuating antigen presentation to CD4 + T-cells^57^. While generally underappreciated, this ability of epithelial cells to function as accessory APCs is highly relevant to anti-tumor immunity^58^. Specifically, MHC-II expression on carcinoma cells likely increases tumor recognition by the immune system^27^. Indeed, tumor specific MHC-II expression is associated with superior prognosis and/or improved response to immune checkpoint inhibitor therapy in multiple cancers, as well as enhanced tumor rejection in mouse models^27,28,59–62^. Recent loss of function and complementation studies in murine carcinoma cells also showed that tumor cell expression of MHC-II is associated with higher Th1 cytokine levels, T-cell infiltration, and sensitivity to anti-PD-1 therapy^63^.

While MHC-II expression has been reported in GCs, most existing studies have investigated only individual isotypes, such as HLA-DR^28,41,42^. Furthermore, cell culture models using established gastric cancer lines have clearly detected MHC-II expression, often in response to IFNγ^39,64^. However, no studies have comprehensively analyzed the mRNA expression status of the entire MHC-II antigen presentation pathway in GCs. In this report, our objective was to determine the impact of EBV on expression of MHC-II components and how this correlated with markers of immune infiltration and activation.

Using data from nearly 400 GCs, we determined that EBVaGCs display high mRNA levels for virtually all MHC-II genes, as well as the MHC-II-like α- and β-chains, and the invariant chain encoded by CD74 (Figs. 1, 2, 3, 5). We concurrently detected high levels of CIITA and RFX5 expression in EBVaGCs, which are key transcriptional regulators of MHC-II pathway genes^26,51^. Significantly higher levels of all these genes were present in EBVaGCs as compared to normal control samples, and these levels were typically higher than those detected in other GCs (Fig. 6). These elevated levels of expression are likely related to the higher levels of IFNγ expressed in EBVaGCs (Fig. 6), which ultimately reflects the T-cell inflamed nature of EBVaGCs^65^. The coordinated upregulation of MHC-II pathway genes by IFNγ is clearly illustrated by the strong global correlations presented in Fig. 7. Our conclusions are supported by a recent single cell RNA sequencing analysis that conclusively shows much higher expression of HLA-DPA1 and HLA-DPB1 mRNAs in malignant epithelial cells isolated from an EBVaGC compared to the EBV-negative GCs they characterized^47^. Single cell RNA sequencing also demonstrated that the EBVaGC malignant epithelial cells exhibited a characteristic enrichment of genes fitting into the gene ontology terms “antigen processing and presentation of peptide antigen via MHC-II” and “interferon-gamma-mediated signaling processes”^47^, precisely mirroring our conclusions based on sequencing of bulk tumor mRNA.

The activation of circulating CD4+ and CD8+ T-cells begins when they encounter their specific cognate antigen presented on either class-II or class-I MHC molecules, respectively^21,22^. Ligation of the antigen-loaded MHC complex with the TCR represents signal 1, and initiates T-cell activation. However, T-cells require a secondary signal via co-stimulatory molecules like CD28 for activation, and avoidance of anergy^20^. Interestingly, EBVaGCs expressed higher levels of the co-stimulatory receptor CD28 versus other GC subtypes and normal control tissues, suggesting increased T-cell infiltration (Fig. 8). Ligation of CD28 with either the CD80 or CD86 ligands leads to clonal T-cell expansion in an antigen-specific fashion^20,56^. This response is attenuated by the subsequent induction of the co-inhibitory molecule CTLA-4, encoded by CD152. CTLA-4 competes with CD28 for binding to either CD80 or CD86 to negatively regulate T-cell responses^20,55^. CTLA-4 is significantly upregulated in EBVaGCs compared to other GC subtypes or normal control tissue, providing further evidence of enhanced T-cell activation in this GC subtype (Fig. 9). Taken together, these data suggest that there are not only a higher number of infiltrating T-cells within the EBVaGC tumor microenvironment, but that T-cells are more likely to have been activated by their cognate antigens.

To persist and survive, fully activated T-cells also require survival signals^20,56^. The receptors that generate these survival signals are expressed only after productive TCR-mediated antigen-specific T-cell activation and/or CD28 co-stimulation^20,56^. Importantly, we determined that EBVaGCs express higher levels of all inducible T-cell survival genes compared to other GCs and normal control tissues (Fig. 9). The increased expression of genes encoding survival signal molecules that are only induced following TCR-mediated antigen-specific T-cell activation and/or CD28 co-stimulation further indicates that increased levels of proliferating and activated T-cells are present in the EBVaGC tumor microenvironment. These results agree with previous reports that more T-cells are present in EBVaGCs compared to other GC subtypes^40,41,66^, but extend these observations further by providing evidence of productive T-cell activation by MHC-II dependent tumor-antigen recognition.

The enhanced coordinate expression of MHC-II pathway components is likely mediated by intratumoral IFNγ. Indeed, IFNγ levels are higher in EBVaGCs than other GC subtypes or normal tissue (Fig. 6). CIITA and RFX5, two important activators of transcription of MHC-II pathway genes that are regulated by IFNγ, are highly upregulated in EBVaGCs and their expression is strongly correlated with IFNG across individual tumor samples (Figs. 6 and 7). This observation was unexpected, as multiple EBV products have been reported to antagonize the IFNγ response. These include the EBV immediate early protein BZLF1^53^ and the EBV latency proteins LMP2A and LMP2B^54^. Clearly, none of these EBV proteins can completely block IFNγ-mediated increases in CIITA, RFX5 and the subsequent coordinate upregulation of transcription of MHC-II pathway genes in the context of these actual human tumors. Furthermore, expression of BZLF1, LMP2A, or LMP2B was not inversely correlated with the levels of CIITA or other components of MHC-II across patient samples (Fig. 7). It is possible that expression of these viral genes is below the threshold needed to effectively antagonize the IFNγ response pathway. Alternatively, these EBV mRNAs might represent contamination from lytically infected B-cells infiltrating the tumor, but this seems unlikely given the general prevalence of EBV infection in the population and the failure to detect them in EBV-negative GCs. Interestingly, EBVaGCs exhibit the strongest signature of IFNγ response across the different GC subtypes, further suggesting that the IFNγ response in these cancers is not effectively blocked by EBV^5,10^. In addition, some EBVaGC derived cell lines have also been shown to maintain an intact IFNγ response pathway, despite expressing various EBV proteins^67,68^.

Importantly, the detection of high mRNA levels for all components of the MHC-II antigen presentation apparatus does not necessarily translate to a high level of expressed protein, correct protein localization, or function in EBVaGCs. However, immunohistochemical analyses of EBVaGCs observed high levels of at least HLA-DR and HLA-DP protein^41,42,47^, and the coordinated regulation of these genes suggests that the other MHC-II molecules will be similarly present. EBV-positive nasopharyngeal carcinomas frequently express high levels of HLA-DR protein as well^69^. Thus, it seems likely that the level of expression of EBV encoded antagonists of MHC-II dependent antigen presentation is not sufficient to grossly impact the MHC-II presentation system in EBVaGCs and perhaps other EBV-associated carcinomas.

In summary, the development of the tumor directed CTL responses necessary to control and clear malignancy initially depends on antigen presentation by MHC-II. The coordinated upregulation of the components of the MHC-II antigen presentation pathway, combined with the expression of exogenous viral antigens may help explain why clinical outcomes are superior for EBVaGCs compared to most other GC subtypes. An analogous situation is likely present in the tumor microenvironment of human papillomavirus positive oropharyngeal cancers, which display similar immunological characteristics to EBVaGCs, including upregulated MHC-II pathway components, and also exhibit better patient outcomes compared to those without a viral etiology^70,71^.

## Materials and methods

### RNA expression comparisons and statistical analysis

Level 3 RSEM normalized RNA expression data for the TCGA/PanCancer Atlas gastric carcinoma (STAD) cohort was downloaded from the Broad Genome Data Analysis Centers Firehose server (https://gdac.broadinstitute.org/). The gene level Firehose dataset was used for all genes. Normalized expression data was imported into Microsoft Excel and GC subtypes were manually curated as described previously^24^. Briefly, patient samples were subdivided into 5 groups based on GC subtype (EBV-associated gastric adenocarcinoma (EBVaGC), chromosomal instability (CIN), genomically stable (GS), or microsatellite instability (MSI)) or classification as normal control gastric tissue, resulting in 30 EBVaGC, 223 CIN, 50 GS, 73 MSI, and 35 normal control samples with data available for gene expression analysis. Expression levels were compared by gastric cancer subtype using Mann–Whitney U tests in RStudio (version 1.2.1335). Derived p-values were corrected for multiple testing using the Benjamini–Hochberg method. The threshold of significance for reported FDR (false discovery rate) values was set at 0.1. Boxplots and heatmaps (Figs. 1, 2, 3, 4, 5, 6, 7, 8, 9) were generated using the ggplot2 package in RStudio (version 1.2.1335) and final figure layouts were performed with CorelDRAW (version X7) as described^24^.

### Quantification of inflammatory infiltrate on histologic sections

Cases of EBVaGC with available high-quality diagnostic histologic slides (n = 24) were downloaded from the TCGA data portal. Slides were analyzed using QuPath^52^. A representative 2 mm^2^ area was assessed in each case that contained > 10,000 detected cells. Inflammatory cells were detected using the standard cell detection function to identify smaller cells (area between 10 and 40 μm^2^) with darkly stained nuclei. Due to differences in hematoxylin and eosin stains between cases the threshold for hematoxylin nuclear stain was varied between 0.3 and 0.6 to optimize selection of darkly stained inflammatory cells. The total cells were then calculated using the same cell detection function to quantify cells with an area between 10 and 40 μm^2^ and a threshold of hematoxylin staining of 0.1. The detected inflammatory cell infiltrate is shown as a percentage of the total cells. All slides and cell detections were reviewed by a pathologist (MJC).

### Correlation matrix

Level 3 RSEM normalized RNA-seq data for the indicated MHC-II pathway genes was extracted and processed for the EBVaGC, CIN, GS, and MSI samples as detailed above. Normalized RNA expression data for the indicated EBV genes in 26 EBVaGC samples was obtained from Chakravorty et al*.*^10^. Pairwise Spearman correlation analysis for each of the indicated genes involved in the MHC-II dependent antigen presentation system or its regulation was performed as before using the ggplot2 package^72^ in RStudio (version 1.2.1335) and final figure layout was performed with CorelDRAW^24^.

## Supplementary information

Supplementary Information 1.
