# Supplementary material for: High MHC-II expression in Epstein–Barr virus-associated gastric cancers suggests that tumor cells serve an important role in antigen presentation
Source: Sci Rep. 2020 Sep 8;10:14786. doi: 10.1038/s41598-020-71775-4 (PMC7479113; doi:10.1038/s41598-020-71775-4)
Supplement: Supplementary file 1 — Supplementary Information 1. [file 41598_2020_71775_MOESM1_ESM.docx]

**Supplementary Data for:**

High MHC-II expression in Epstein-Barr Virus-associated gastric cancers suggests that tumor cells serve an important role in antigen presentation.

**Authors:**

Farhad Ghasemi^1*^, Tanner M. Tessier^2*^, Steven F. Gameiro^2*^, Allison H. Maciver^1,3^, Matthew J. Cecchini^4^, and Joe S. Mymryk^2,3,5,6^

**Author’s Affiliation:**

^1^Department of Surgery, Western University, London, ON, Canada N6A 4V2.

^2^Department of Microbiology and Immunology, Western University, London, ON, Canada N6A 3K7.

^3^Department of Oncology, Western University, London, ON, Canada N6A 3K7.

^4^Department of Pathology and Laboratory Medicine, Western University and London Health Sciences Centre, London, Ontario, Canada N6A 5C1.

^5^Department of Otolaryngology, Head & Neck Surgery, Western University, London, ON, Canada N6A 5W9.

^6^London Regional Cancer Program, Lawson Health Research Institute, London, ON, Canada N6C 2R5.

*these authors contributed equally

**Corresponding author:**

Joe Mymryk

London Regional Cancer Program

Room A4-837, 790 Commissioners Rd. East

London, Ontario, Canada N6A 4L6

Phone: (519) 685-8600 ext. 53012 Fax: (519) 685-8616

Email: [jmymryk@uwo.ca](mailto:jmymryk@uwo.ca)


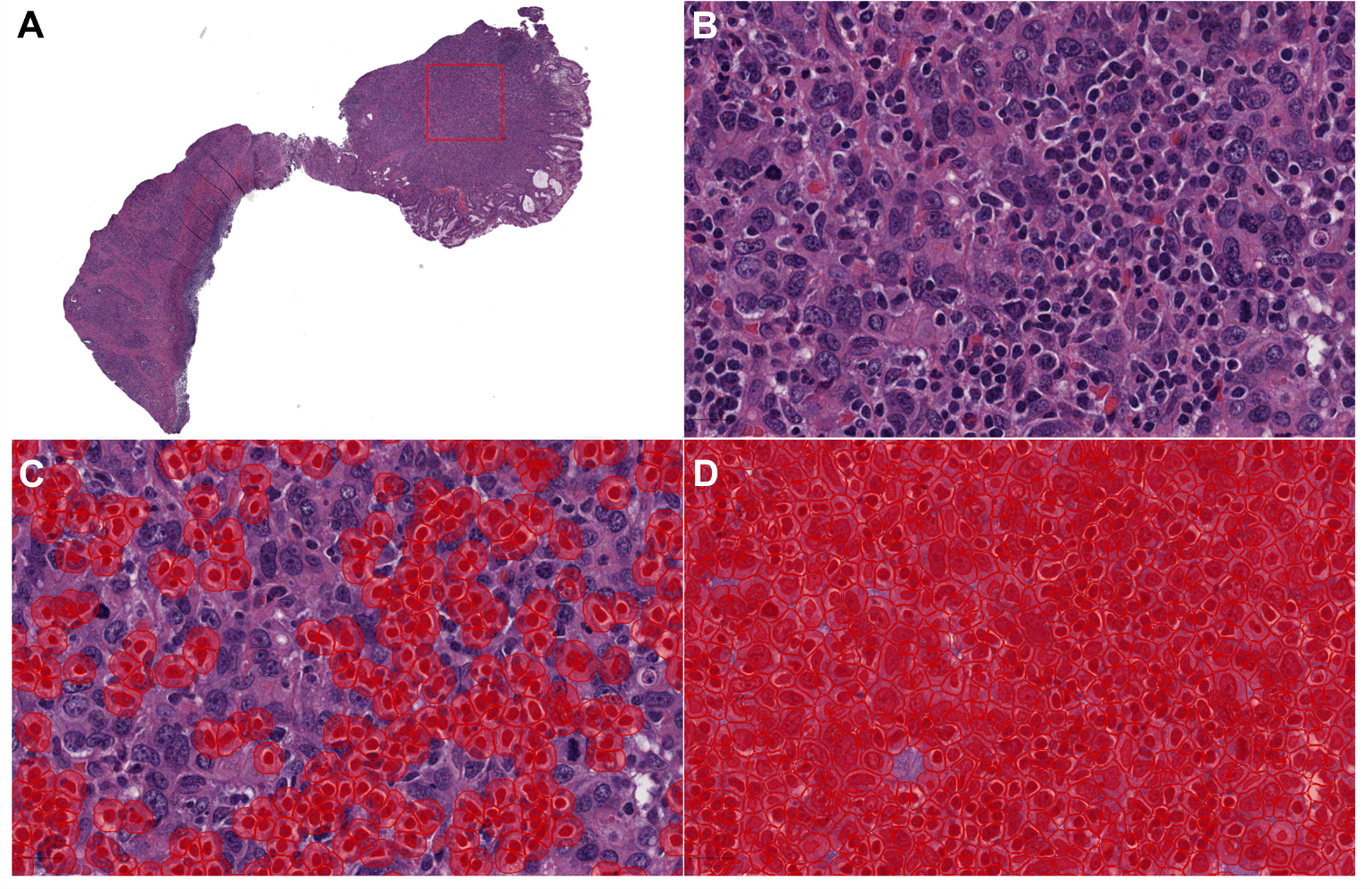


**Supplemental Figure S1: Quantification of inflammatory infiltrate on histologic sections.** (A) Low power photomicrograph (magnification 10x) of an EBV-related gastric carcinoma with a representative 2 mm^2^ area denoted (red square). (B-D) High power photomicrographs (400x magnification) of (B) an EBV related gastric carcinoma with neoplastic tumor cells and abundant inflammatory cell infiltrate, (C) Detected small darkly stained inflammatory cells highlighted with red shading, and (D) total detected cells highlighted by red shading. Figure was prepared with CorelDRAW (version X7).

**Supplemental Table S1: Inflammatory cell infiltration scores and detection settings**

| Sample Barcode | Inflammatory Cells | Total Cells | % Inflammatory | Nuclear Intensity Threshold | Min Nuclear Area (µm^2^) | Max Nuclear Area (µm^2^) |
| --- | --- | --- | --- | --- | --- | --- |
| TCGA-BR-6455-01 | 2106 | 21017 | 10.02 | 0.4 | 10 | 40 |
| TCGA-BR-6706-01 | 4655 | 19012 | 24.48 | 0.3 | 10 | 40 |
| TCGA-BR-6707-01 | 4224 | 21167 | 19.96 | 0.3 | 10 | 40 |
| TCGA-BR-7196-01 | 2707 | 11860 | 22.82 | 0.35 | 10 | 40 |
| TCGA-BR-7958-01 | 5555 | 20574 | 27.00 | 0.5 | 10 | 40 |
| TCGA-BR-8366-01 | 2012 | 13497 | 14.91 | 0.35 | 10 | 40 |
| TCGA-BR-8381-01 | 3741 | 18854 | 19.84 | 0.5 | 10 | 40 |
| TCGA-BR-8589-01 | 8471 | 26771 | 31.64 | 0.55 | 10 | 40 |
| TCGA-BR-A4J4-01 | 2923 | 13675 | 21.37 | 0.4 | 10 | 40 |
| TCGA-CD-5801-01 | 4738 | 20536 | 23.07 | 0.6 | 10 | 40 |
| TCGA-D7-5577-01 | 4198 | 20015 | 20.97 | 0.4 | 10 | 40 |
| TCGA-D7-8570-01 | 10394 | 24749 | 42.00 | 0.4 | 10 | 40 |
| TCGA-D7-8573-01 | 1956 | 12849 | 15.22 | 0.3 | 10 | 40 |
| TCGA-D7-A4YX-01 | 4123 | 18719 | 22.03 | 0.4 | 10 | 40 |
| TCGA-D7-A6EZ-01 | 2384 | 18357 | 12.99 | 0.45 | 10 | 40 |
| TCGA-FP-7916-01 | 3181 | 17401 | 18.28 | 0.5 | 10 | 30 |
| TCGA-FP-7998-01 | 6673 | 21331 | 31.28 | 0.5 | 10 | 40 |
| TCGA-HU-8608-01 | 6811 | 21206 | 32.12 | 0.45 | 10 | 40 |
| TCGA-HU-A4G2-01 | 5835 | 16541 | 35.28 | 0.45 | 10 | 40 |
| TCGA-HU-A4H0-01 | 5122 | 16800 | 30.49 | 0.5 | 10 | 40 |
| TCGA-VQ-A8PF-01 | 4348 | 21956 | 19.80 | 0.6 | 10 | 40 |
| TCGA-VQ-A91W-01 | 1397 | 18587 | 7.52 | 0.6 | 10 | 40 |
| TCGA-VQ-A923-01 | 4231 | 19346 | 21.87 | 0.55 | 10 | 40 |
| TCGA-VQ-AA69-01 | 3336 | 17781 | 18.76 | 0.6 | 10 | 40 |

**Supplemental Table S2: Correlations between inflammatory cell infiltration score and expression of genes involved in the MHC-II antigen presentation pathway in EBVaGCs**

| Gene | Rho | p-value | FDR |
| --- | --- | --- | --- |
| HLA-DMA | 0.577 | 0.004 | 0.045 |
| HLA-DMB | 0.517 | 0.011 | 0.045 |
| HLA-DOA | 0.49 | 0.016 | 0.045 |
| HLA-DOB | 0.296 | 0.160 | 0.182 |
| HLA-DPA1 | 0.457 | 0.026 | 0.063 |
| HLA-DPB1 | 0.356 | 0.089 | 0.128 |
| HLA-DQA1 | 0.354 | 0.090 | 0.128 |
| HLA-DQA2 | 0.509 | 0.012 | 0.045 |
| HLA-DQB1 | 0.179 | 0.401 | 0.426 |
| HLA-DQB2 | 0.429 | 0.038 | 0.078 |
| HLA-DRA | 0.498 | 0.014 | 0.045 |
| HLA-DRB1 | 0.374 | 0.073 | 0.124 |
| HLA-DRB5 | 0.304 | 0.148 | 0.180 |
| HLA-DRB6 | 0.55 | 0.006 | 0.045 |
| CD74 | 0.344 | 0.100 | 0.131 |
| CIITA | 0.422 | 0.041 | 0.078 |
| RFX5 | 0.136 | 0.526 | 0.526 |

Spearman’s correlation analysis between inflammatory cell score and mRNA expression of the indicated MHC-II pathway genes in EBVaGC. Spearman’s rank correlation coefficient (Rho), p-values and FDR for each comparison are indicated. Statistical analysis was done RStudio (version 1.2.1335). Highlighted rows indicate statistically significant correlations.
